# The Use of Mumsnet by Parents of Young People With Mental Health Needs: Qualitative Investigation

**DOI:** 10.2196/18271

**Published:** 2020-09-03

**Authors:** Lauren Croucher, Elif Mertan, Roz Shafran, Sophie D Bennett

**Affiliations:** 1 University College London Great Ormond Street Institute of Child Health London United Kingdom

**Keywords:** mental health, parenting, internet, evidence-based medicine

## Abstract

**Background:**

There are high rates of mental health needs in children in the United Kingdom, and parents are increasingly seeking help for their children's needs. However, there is not enough access to child and adolescent mental health services and parents are seeking alternative forms of support and information, often from web-based sources. Mumsnet is the largest web-based parenting forum in the United Kingdom, which includes user-created discussions regarding child mental health.

**Objective:**

This qualitative investigation aimed to explore the emergent themes within the narratives of posts regarding child mental health on Mumsnet and to extrapolate these themes to understand the purpose of Mumsnet for parents of children and young people with mental health needs.

**Methods:**

A total of 50 threads from Mumsnet *Talk Child Mental Health* were extracted. Following the application of inclusion and exclusion criteria, 41 threads were analyzed thematically using the framework approach, a form of qualitative thematic analysis.

**Results:**

In total, 28 themes were extracted and organized into 3 domains. These domains were *emotional support*, *emotional expression*, and *advice and information*. The results suggested that parents of children with mental health needs predominantly use Mumsnet to offer and receive emotional support and to suggest general advice, techniques, and resources that could be applied outside of help from professional services.

**Conclusions:**

This paper discusses the future of health information seeking. Future research is required to establish initiatives in which web-based peer-to-peer support and information can supplement professional services to provide optimum support for parents of children with mental health needs.

## Introduction

### Background

Recent years have seen a slight but notable increase in the prevalence of mental health disorders in children over time in the United Kingdom, 9.7% of those aged 5-15 years with a diagnosable condition in 1999, to 11.2% in 2017 [1]. More recently, in 2018, a separate survey noted that 12.8% of those aged 5-19 years in the United Kingdom met the clinical criteria for a mental health disorder [2]. The demand for Child and Adolescent Mental Health Services (CAMHS) has also risen significantly [3] as awareness of mental health and help-seeking behaviors for childhood mental health conditions are increasing [4,5].

Fortunately, there are effective treatments for childhood mental health disorders [6]. However, in the United Kingdom, approximately 1 in 4 young people referred to mental health services were refused treatment in 2018 to 2019 [7], and 1 in 5 young people with a disorder reported a waiting time of over 6 months to receive specialist assistance [1]. Survey data have suggested that during this wait for CAMHS, two-thirds of parents report not being referred to or directed toward (signposted to) appropriate forms of support [8]. Parents and young people are therefore turning to alternative sources of information and support, such as the internet [9,10]. In 2018, 89% of adults in Great Britain used the internet at least weekly [11], and user-driven online discussion fora (web-based discussion in which the content is solely created by the public using the site) for health-related concerns have become increasingly popular [12]. This investigation aims to understand the use of an existing popular user-driven web-based discussion forum (Mumsnet) by parents of children with mental health needs.

To date, no research has been conducted on the role of web-based fora for parents of children with mental health needs. Nonetheless, the purpose of user-driven web-based communities in relation to other health needs has been qualitatively examined by establishing emerging themes within the web-based narratives. Scharett and colleagues [13] qualitatively investigated the purpose of web-based peer support groups for caregivers of people with Alzheimer disease. This investigation suggested that the web-based group provides a platform for caregivers to discuss their confusion over symptoms of the disease and highlights the necessity for emotional support among caregivers. This analysis quantitatively explored the emergent themes and found that 59.6% (149/250) of posts contained emotional content in which the blog was used as an opportunity for emotional expression, for example, “She will sob and cry... which is heart-breaking to us....” Furthermore, 40.16% (98/244) of the responses on the blog provided informational support to caregivers, with 21.31% (52/244) of posts advising other caregivers to seek professional support. Web-based communities for caregivers of people with Alzheimer disease therefore not only provide a platform for information seeking but also create a space for emotional regulation.

Similar lessons can be learned from research regarding the use of web-based platforms for other health needs by young people and parents. Kirk and Milnes [14] examined 151 discussion threads on a cystic fibrosis charity website and discovered 5 emergent themes. These themes included managing treatments, managing emotions, managing relationships, managing identity, and managing support. The web-based community provided a liminal ground between the geographically distant users and their similar experiences, which was particularly important when considering young people with cystic fibrosis cannot make physical contact because of the risks of cross-infection [14]. Therefore, web-based platforms can benefit users by allowing those who may not be able to meet in person the opportunity to share experiences and advice with others.

Widemalm and Hjärthag [15] qualitatively investigated the use of web-based fora by children of parents with mental health needs. The investigation revealed 4 emergent themes. The first of these themes was caregiver burden in which the young people expressed the pressures they faced caring for their parents. The second theme was knowledge seeking within which users sought information from other users on the site. Third, support from the forum was a common theme in which users felt confident in discussing their issues in the virtual platform. This was apparent for stigmatizing illnesses that users did not feel comfortable discussing outside of a web-based capacity. In addition, users appreciated the ability to discuss their issues anonymously and interact with people who were experiencing similar issues. Finally, frustration and powerlessness over health care were often expressed by young people. The web-based community, established to discuss mental health, provided users with the opportunity to seek social support in a safe environment and gather information about their parents’ mental health needs.

The combination of a lack of access to services and the increasing popularity of web-based platforms leaves one questioning the future landscape of mental health support and information for parents of children with mental health needs. Could this popular and accessible method of web-based information and support be proliferated to help parents in need, especially those who desperately require support and information while on CAMHS waiting lists? As no research to date has been conducted on the role of web-based fora for parents of children with mental health needs, exploring the emerging themes within web-based fora for parents could aid our understanding of the reasons why parents of children with mental health needs use these sites. Furthermore, such research could provide evidence as to whether web-based platforms may be used as an adjunct and/or alternative to traditional services.

### Aims and Objectives

In summary, there are high rates of mental health problems in young people in the United Kingdom and an increase in help-seeking behaviors for these disorders. Despite effective treatments for these disorders, there is not enough access to CAMHS. Parents are increasingly turning to web-based methods of communication and information seeking, and many studies have highlighted not only the benefits of posting but also the emergent themes within web-based narratives about health care. However, there is a lack of research investigating the reasons why parents of children with mental health needs are turning to web-based sources of information or support seeking. This study aimed to explore the role of an internet forum for parents of young people with mental health needs (Mumsnet). Mumsnet is the largest parenting forum in the United Kingdom [16], which is used by 14% of the UK population and has received over 3.9 billion views in the last 10 years [17]. The research used qualitative thematic analysis, which is not dictated by a hypothesis [18]. The specific objectives were as follows: (1) to explore the emergent themes within the narratives of posts on the web-based forum and (2) to extrapolate these themes to understand the purpose of the web-based forum for parents of children and young people with mental health needs.

## Methods

### Study Design and Data Analysis

The study used qualitative methods outlined in Braun and Clarke [19] to investigate the reasons why parents of children with mental health needs posted on Mumsnet. The analysis was based on the methodology of studies investigating Twitter, blogs, and web-based fora [20,21].

### Mumsnet

This investigation analyzed posts on the Mumsnet discussion forum *Talk*. Mumsnet *Talk* offers an open platform for discussions organized around over 200 established topics, known as threads, and receives over one million visits per month [22]. The Mumsnet *Talk* discussion forum can be viewed by any internet user, but only Mumsnet account holders can participate in the discussion. This study analyzed content posted under the *Talk* discussion page titled *Child Mental Health*. As of June 6, 2019, there were more than 900 active discussions under the *Child Mental Health* section.

### Ethics

The study received University College London (UCL) ethical approval (ref: 15209/001) and Research and Development approval from the UCL Joint Research and Development Office (19PP05). In line with the recommendations of the Association of Internet Researchers Ethics Working Committee [23], all data were extracted without the inclusion of usernames, and direct quotes were altered slightly (without changing meaning) to maintain the privacy of those posting on the forum during the initial data extraction stage of the analysis.

### Data Collection

The most recent threads of the *Talk:* *Child Mental Health* heading were extracted to capture up-to-date narratives posted on the site. In line with qualitative research guidelines [24], data extraction was complete once the analysis reached data saturation, suggesting all possible themes within the chosen narratives were discovered. The initial data set consisted of 50 threads that contained over 750 posts. The threads dated from February 4, 2019, to June 6, 2019.

### Inclusion and Exclusion Criteria

**Figure 1:**
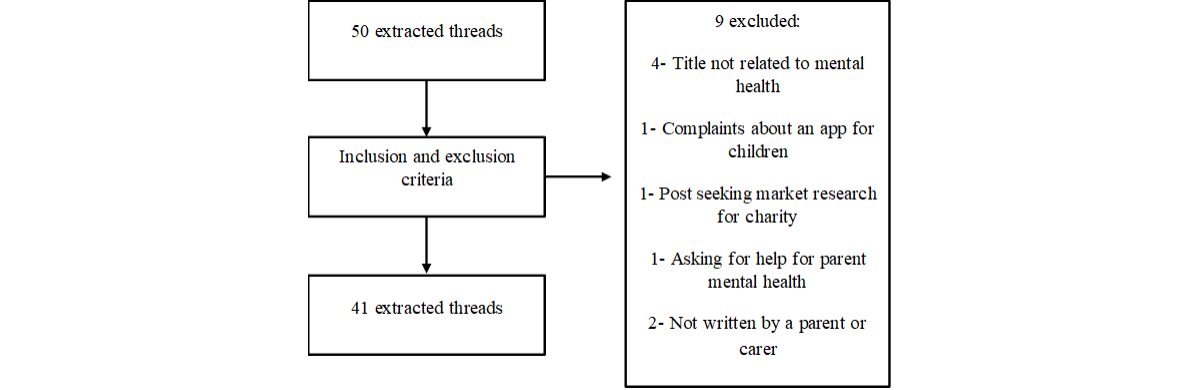
Flow chart of eligible threads and reasons for exclusion.

Only posts or comments within a thread in which the original post was directly related to child mental health were considered, and posts discussing child physical health or school attainment were excluded from the analysis. Posts had to be written by a person who identified as a parent or carer of a child with a mental health need, rather than professionals, as far as investigators could establish from reading the posts. The investigators excluded threads in which the original message was posted by agents other than parents or carers such as businesses seeking marketing research or professional mental health workers. Figure 1 shows a flowchart of eligible threads and reasons for exclusion.

### Data Extraction

Parsehub was used to extract data from Mumsnet threads. Parsehub is a freely available web-based scraping tool designed to extract internet data. Any original posts or comments including information that could potentially identify the user, such as age, name, or location, were omitted manually by the researchers before the data were analyzed. Following this, the raw data were transferred into word documents for analysis.

### Qualitative Analysis of Posts

All data were analyzed using the framework approach [19], a form of qualitative thematic analysis. All threads were read several times by the principal researcher. Key themes were then highlighted within the text, and notes were made within the margins of the raw data where applicable. The notes were used to create themes within the threads that, in line with qualitative research principles, were grounded in the data and established from users’ own words [25]. These initial themes from all threads were reviewed to ensure that the themes were distinct and sensible, in line with recommendations from a study by Maguire and Delahunt [26]. Themes were then synthesized into a single document for subsequent analysis. The themes and subthemes were synthesized into a code framework. The framework was revisited and revised where necessary, and in line with the principles of qualitative research [27], a manageable index of 20 themes was created from the synthesized original codes. The framework was then applied to the original data [27]. This allowed the researcher to establish whether all themes within the framework were relevant. This enabled the consistency of the themes to be established, and any neglected codes were added. Links were identified within the themes that were then synthesized to establish 3 domains. Thematic charts highlighting examples of each domain and themes within the domains were created. The reliability of the codes was tested by another member of the research team (EM) using the formula described by Miles and Huberman [28]. The second researcher was required to apply the codes established from the final framework to a random selection of 8 threads (20% of the final data set). The threads were chosen using a random number generator, and the second researcher blind coded the threads into the categories they deemed appropriate. There is debate within the field regarding the efficacy of interrater reliability in qualitative research [29]; thus, there is no established threshold within the literature determining an acceptable level of agreement between coders [30]. The researchers agreed that any interrater reliability percentage of 70% or above was acceptable for this methodology, based on the Cohen substantial 0.61 to 0.80 kappa statistic [31]. There was 70% agreement between coders for the nature of codes within the data set. The remainder were resolved by discussion with RS and SB.

## Results

### Themes Identified

Following the application of the inclusion and exclusion criteria, 9 threads were excluded, and 41 threads were deemed eligible for analysis. The flowchart of the eligible threads is displayed in Figure 1. The characteristics of each included thread according to featured mental health needs are displayed in Table 1.

A total of 28 themes were highlighted within the selected threads on Mumsnet *Talk*. These themes were organized into 3 domains: *emotional support*, *emotional expression*, and *advice and information*. The 28 themes are explored below, supported with extracts of Mumsnet *Talk* posts. Multimedia Appendix 1 visually displays the themes and subthemes within the posts.

**Table 1:** The number of threads discussing each mental health need.

Mental health need	Number of threads
Anxiety	10
Multiple	6
Undiagnosed	6
Depression	5
Self-harm	3
Not stated	3
Autistic spectrum condition	2
Attention-deficit hyperactivity disorder	2
Intrusive thoughts	1
Transgender	1
Other	1
Obsessive compulsive disorder	1

### Domain 1: Emotional Support

Content grouped within this domain demonstrated Mumsnet users expressing a need for support or offering support.

#### In Need of Support

Of 41 selected threads, 10 (24%) expressed a lack of or need for support:

I don’t have any support with this.

Thread 5, page 3

I have been looking for a support thread.

Thread 5, page 7

#### Web-Based Community

Of 41 analyzed threads, 10 (24%) included elements of the web-based community on Mumsnet. This included expressions of camaraderie between users:

You are amongst friends here.

Thread 5, page 34

This also included posts in which users updated and kept in touch with each other via the forum by asking questions:

I was just wondering how you are getting on?

Thread 3, page 6

Furthermore, posts within this category included instances comparing the use of the web-based community with discussing issues in person:

I’m not really talking to real people about this.

Thread 5, page 34

#### Offering Emotional Support

The largest category within the emotional support domain was offering emotional support, which featured in 21 of the 41 posts (51%). Posts within this category included comments in which Mumsnet users offered emotional support to other users. This was evident in the form of empathy and sympathy:

So sorry you’re feeling it too

Thread 5, page 4

This was also evident as Mumsnet users discovered that they were not alone in their struggles:

You are not alone.

Thread 38, page 3

Furthermore, emotional support was offered through well wishes and encouragement:

Know that things CAN get better.

Thread 28, page 2

In addition, praise for the parents by other Mumsnet users provided emotional support:

Your son is fortunate to have you on side and it’s great that you’re seeking advice.

Thread 34, page 2

#### Sounds Like Me or My Child

Of 41 threads, 13 (32%) featured emotional support when users highlighted the similarities between their own and another user’s child. Parents shared comments that allowed Mumsnet users to normalize their experiences through learning that other mothers are experiencing similar issues:

He sounds very similar to my son.

Thread 1, page 13

### Domain 2: Emotional Expression

The second domain centered around parents using Mumsnet as a platform to express their emotions.

#### Desire to Write About Experiences

Of 41 threads, 5 (12%) featured expressions of a desire to write about experiences:

I'm just going to use this as a place to vent and write everything down.

Thread 10, page 4

#### Struggling to Cope

The most frequent theme within the emotional expression domain was struggling to cope, which featured in 16 of the 41 threads (39%). Within this theme, Mumsnet users professed their difficulties in managing their child’s mental health problems:

I’m struggling to help him.

Thread 36, page 1

#### Guilt

This theme encompassed comments in which users blamed themselves for their children’s issues or expressed feelings of guilt about their child’s condition. This theme was apparent in 9 of the 41 selected threads (22%):

I do blame myself – have I broken her?

Thread 34, page 6

#### Frustrated With the Situation

Parents often used the discussion thread as a platform to express their frustrations with the situation they were facing. This theme was apparent in 5 of the 41 threads (12%). This included posts discussing comparisons with physical health issues, expressing a desire for their child to be 'normal' and wishing for solutions to their children’s complex problems:

Cannot face it anymore, can't face talking to him, just want him to be normal.

Thread 5, page 19

#### Desperation

Of 41 threads, 7 (17%) featured users’ expressions of desperation as their situation escalated or worsened:

I’m posting out of desperation.

Thread 36, page 1

#### Parent Mental Health

Posts within this theme mentioned the users’ own mental health issues. This theme was found in 7 out of 41 threads (17%):

I have been to the GP very recently because I was too stressed with it all.

Thread 5, page 5

This theme also included posts in which users encouraged other users to seek help for their own mental health:

It's also important to look after your own mental health.

Thread 34, page 6

#### Fear

The second most common theme within the emotional expression domain was fear and worry, which occurred in 13 of the 41 threads (32%):

I'm absolutely terrified for my son.

Thread 11, page 1

#### Confusion

Mumsnet users also used the forum to discuss their confusion regarding certain issues. This theme was apparent in 5 of the 41 threads (12%):

What’s OCD, what’s ASD and what is typical teenageryness. It’s impossible to untangle it all.

Thread 5, page 27

### Domain 3: Advice and Information

Mumsnet users often used the discussion forum to not only receive but also offer advice and information on certain problems.

#### Sharing and Offering Advice and Information

##### Personal Experiences

###### Complaints

Of 41 threads, 15 (36%) included complaints about services. These complaints were grouped into 3 broad categories.

####### Issues With Access

Mumsnet users used the site to complain about accessing mental health services:

My DS has been referred to CAMHS, but as we all know, it can be a long wait.

Thread 16, page 1

It seems ridiculously hard [to find a specialist].

Thread 4, page 1

####### Issues With Help Received

Mumsnet users also complained about the help they had received from mental health services:

The clinician was so disorganised, patronising and incompetent, he ended up making our son's problems worse.

Thread 20, page 3

####### Issues With the System Generally

Mumsnet users also complained more generally about the mental health system:

The underfunding has caused a huge mess of the service and this is where the anger should be directed.

Thread 20, page 10

###### Praise

The forum was also used as an opportunity to praise services. This theme occurred in 7 of the 41 threads (17%):

All professionals/practitioners are supportive, nice people.

Thread 5, page 41

###### In My Experience as a Child

Mumsnet users would often share stories from their own childhoods to allow other users to understand their children’s situations. This theme was found in 9 of the 41 threads (22%):

I suffered from horrible nausea and anxiety at the same age as your child.

Thread 28, page 4

###### In My Experience With My Child

Of the 41 threads, 14 (34%) featured instances of users offering advice to others by sharing what worked with their own child who was experiencing similar problems:

It helped us all to take the Psychiatrist’s advice to speak about the OCD as something other than our son and not lump them together.

Thread 5, page 49

##### Advice

###### Suggesting Resources and Techniques

The most frequently found theme within the advice and information domain was suggesting resources and techniques, which occurred in 21 of the 41 selected threads (51%). This theme incorporates instances in which parents suggested techniques, resources, or general advice on how to help their children, outside of help from professional services:

Look at gentle parenting sites to help him.

Thread 11, page 3

Often, parents would signpost other parents to appropriate resources:

Hi there are some useful resources here [shares website].

Thread 29, page 1

###### Suggesting Services

The second most common theme within the advice sharing domain was suggesting services, which occurred in 18 of the 41 threads (44%):

Ask your gp for a referral to your local camhs service.

Thread 40, page 1

###### Sharing Advice on How the System Works

Of 41 threads, 11 (27%) featured instances of users providing practical advice to other Mumsnet users on how to get appropriate support within various mental health services:

This is what I've found helps: Read up before your meetings (e.g. nice guidelines) and don’t fill gaps with more words.

Thread 5, page 53

##### Sharing Information and Knowledge

As well as offering advice, users often share knowledge about childhood or certain disorders to enable other users to better understand their situation. This theme was found in 15 of the 41 threads (37%):

The NHS suggest that all children be given a vitamin d supplement.

Thread 22, page 5

##### Sharing Opinion on Case

Of 41 threads, 11 (27%) included occasions in which Mumsnet users shared their opinion on the original post. This theme incorporates instances in which users endeavor to aid the primary poster in establishing a diagnosis or reasons for the behavior in the presented case:

Do you think there's any chance she could be on the autistic spectrum?

Thread 15, page 3

#### Asking for Advice and Information

##### Advice Seeking

The most common help-seeking theme was seeking advice, which occurred in 19 of the 41 threads (46%). This was achieved by asking direct questions or asking for advice from other Mumsnet users:

Anybody got any thoughts on how I help and support him?[Thread 30, page 1]

##### Do Not Know What to Do

Of 41 threads, 13 (32%) included instances of Mumsnet being used as a platform for users to express that they did not know what to do:

I don't know how to help him.

Thread 31, page 1

##### Looking for Services and Resources

Of 41 threads, 9 (22%) depicted parents asking for specific services and resources:

Can anyone suggest how to find a consultant who specialises in ADHD in children and who will see patients privately.

Thread 4, page 1

##### Looking for Extra Services, Resources, or Techniques

Of 41 threads, 6 (15%) contained instances of parents asking for extra services or resources while waiting to receive specialist help:

We are waiting for CAMHS appointment now as life for our family has gotten so intense because of this. In the mean-time I am looking for a parenting course.

Thread 15, page 11

This theme also incorporated instances in which users had completed certain interventions with a service and were looking for extra support:

He’s recently just finished a course of therapy for his anxiety in CAMHS, so we’re not too sure who to turn to.

Thread 3, page 1

##### Looking for Similar Experiences

Of 41 threads, 9 (22%) showed Mumsnet users expressing a desire to hear stories from other parents who were experiencing similar situations:

Any thoughts on what I should do from those who might have been through this?

Thread 5, page 56

##### Should I Be Worried?

Of 41 analyzed threads, 3 (7%) displayed how Mumsnet was used as a platform for parents to seek reassurance or guidance from other users by asking whether they should be concerned about their child’s behaviors:

Should I be worried about her?

Thread 12, page 1

##### Questions About Services or Resources

As well as providing a platform to seek resources, the Mumsnet discussion forum also allowed users to ask questions about specific services or resources. This theme occurred in 7 of the 41 (17%) selected threads:

Has anyone's child been offered CBT (or DBT) through CAMHS/NHS? If so what was it for? How did they get it?

Thread 41, page 1

## Discussion

### Principal Findings

This qualitative investigation aimed to explore the role of a web-based forum for parents of young people with mental health needs (Mumsnet). To our knowledge, this is the first investigation of web-based fora by parents of children with mental health needs. The main domains derived from the narratives were *emotional support*, *emotional expression*, and *advice and information*. Overall, the results suggest that parents of children with mental health needs predominantly use Mumsnet to offer and consequently receive emotional support and to suggest general advice, techniques, and resources that could be applied outside of help from professional services. This suggests a potential role in supporting parents who may not be able to access traditional CAMHS services, those on waiting lists for services, and/or those already accessing services.

The most common form of assistance featured on Mumsnet was offering and receiving emotional support, which featured in 21 of the 41 threads (51%). This is consistent with the research by Widemalm and Hjärthag [15], which examined the use of online fora for young people caring for parents with serious mental health needs. Widemalm and Hjärthag [15] suggested that social isolation caused by caring for someone with a mental health disorder is eased by discussing the situation on web-based platforms. Furthermore, in survey research relating to the use of a web-based mental health site by young people experiencing their own mental health issues, it has been suggested that befriending others with similar realities assures people they are not alone [32]. This research has extended the literature on the formation of social support on web-based fora by young people by highlighting the utility of web-based platforms for facilitating the exchange of social support for parents of children with mental health needs.

Of 41 threads, 10 (24%) embodied the nature of a web-based community, including instances of updating other users and expressing statements of camaraderie. This extends previous research on Mumsnet, highlighting the community nature of the forum through a questionnaire survey and discourse analysis of discussions related to belonging on the site [22]. Mumsnet can be used as a platform for the development of social support, which may be difficult to ascertain in real life because of the burdens associated with caring for a child with mental health needs [33] and a lack of support from professional services. Therefore, as findings have suggested that parents of children with mental health needs seek social support, services should endeavor to signpost parents toward local support groups before, during, and after interventions. Alternatively, it is possible that parents could be signposted to these discussion fora as they have been suggested to provide valuable support.

Although this research has suggested that social support can be meaningfully sought and received on web-based platforms, one must be cautious as, in line with previous research [13], a key theme within the narratives was information sharing and receiving. Discussions on Mumsnet provide parents with general advice and information that could either supplement information provided by professional services or help while families are waiting to receive input from those services. One must be mindful of the quality of information posted on web-based platforms, particularly the extent to which this is evidence based. This research question is being examined in a separate investigation on the same dataset.

Parents expressed concerns regarding not only their inability to help their children but also their confusion over where to look for help. As a lack of knowledge regarding child mental health conditions is a significant barrier to seeking professional help [34], Mumsnet may act as a platform to enable parents to gain advice and information to disseminate barriers to seeking professional help. This suggests that services could create informational resources that are catered to the needs of users of internet support groups to be shared on the sites to increase mental health literacy and confidence in help seeking.

Many posts included discussions about services. A proportion included parents asking direct questions about specific resources and services (7/41, 17%), some included information on how to access specialist care (11/41, 27%), some included providing service recommendations (18/41, 44%), and others asked for service recommendations (9/41, 22%). This suggests that Mumsnet serves as a means to gather information about services, rather than being a replacement for CAMHS and other services. This finding supplements previous research, suggesting parents want information about how CAMHS works before their first appointment [35]. Posts were more likely to request and offer information and advice regarding resources and techniques outside of professional services (21/41, 51%) than recommending services (18/41, 44%). This is contrary to previous research investigating diabetes fora suggesting the most common form of advice offered on web-based platforms is signposting to professional services [36].

Of 41 threads, 15 (36%) in the discussion forum contained a complaint about mental health services, compared with 17% containing praise. This contradicts a previous cross-sectional study examining parent and child satisfaction with CAMHS using a questionnaire [37], suggesting that more than three-quarters of parents and adolescents were either mostly or very satisfied with their experiences with CAMHS. However, McNicholas et al [37] sent the 8-item Client Satisfaction Questionnaire to open cases only and therefore did not include the views of those who had left the service due to dissatisfaction, those who were on waiting lists, or those not able to access services, something which this study was able to consider. A total of 63 complaints were found within the threads that were grouped into 3 broad categories: issues with access, issues with help received, and issues with the system generally. Most complaints within the threads were too general to make any inferences about specific difficulties with existing services, for example, “mental health services are a shambles” (Thread 19). Nevertheless, it must be noted that services were also praised within the threads, for example, “I can vouch for the fact that every member of CAMHS staff I have dealt with has been lovely, professional, helpful” (Thread 20). Future research should endeavor to explore the potential motivators for using web-based fora to criticize as opposed to praise services.

Parents offered personal experiences from looking after their child in 14 of the 41 threads (34%) and offered personal experiences from their own childhoods in 9 of the 41 threads (22%). This supports the suggestion that personal expertise shared on web-based platforms is expressed through stories from people who have experienced certain situations that are more accessible to others [38]. Offering support and sharing experiences, advice, and recommendations were major themes within the narratives on Mumsnet. This supports research by Anderson and colleagues [20], suggesting that altruism is apparent in web-based caregiver communities.

Of 41 threads, 7 (17%) included examples of parents using Mumsnet when their child’s condition had escalated, which adds a new domain to previous research suggesting people seek web-based support at a critical period in their experiences of mental health issues [39]. It may also be apparent that parents seek web-based help during a critical period in their own ability to cope with their child’s needs. Another 7 of the 41 threads (17%) in this study mentioned the mental health problems faced by parents. This extends previous research, which suggested that children of parents with mental health needs discuss their own mental health needs on web-based platforms [15,40]. This analysis displayed instances not only of parents expressing their own mental health struggles but also instances of other users encouraging Mumsnet users to seek help for their own well-being. This suggests that Mumsnet could act as an additional platform that parents can access during a critical time in their experiences. As a web-based source, Mumsnet is constantly accessible, and support within the site is not restricted to opening hours.

On sites such as Facebook, users are conscious of revealing concerns around stigmatizing illnesses because of the identifiable nature of the site [41,42]. In contrast, Mumsnet *Talk:* *Child Mental Health* featured thousands of posts regarding potentially stigmatizing concerns. Previous research suggests that parents fear judgment from health care professionals [43], and this was supported within this research through statements such as, “I’m worried that the doctor will laugh at me” (Thread 38) and “I don't want to go to the GP if it's basically my fault” (Thread 39). Anonymity on web-based platforms encourages self-disclosures [44]. Mumsnet may therefore provide an anonymous space that could act as an alternative to face-to-face services within which parents of children with mental health needs can comfortably express their concerns.

### Limitations

This study is not without its flaws. There is a lack of demographic data available about Mumsnet users because of both the anonymized nature of the site and ethical restrictions that required any identifiable information such as age or occupation to be removed. Thus, inferences cannot be made about whether sample characteristics may have influenced the results. Nonetheless, Mumsnet census data report that the majority of its users are middle-class, university-educated women, and 74% of users have a household income of over the national average [45]. Therefore, suggestions from this research may not be generalizable to the wider population. In addition, as the extracted data could not include the pseudonyms used by parents, it is not possible to know whether all instances of some themes were posted by separate users, as opposed to one, vocal Mumsnet user. Finally, this research cannot be generalized to create inferences about the needs of all parents of children with mental health needs. The results may be specific not only to those with internet access but also those who already actively use Mumsnet in the United Kingdom. It should also be acknowledged that the use of web-based data restricts researchers’ ability to influence conversations, which, although providing an insight into the spontaneous interactions between parents, does not allow the researchers the ability to inquire about comments and extrapolate certain themes.

### Directions for Future Research

As discussed, interventions and resources could be created to cater to the needs of the members of Mumsnet, given the site’s vast popularity and influence among British mothers [22]. Furthermore, parents of children with mental health needs could be signposted toward the site to receive support in the interim period between seeking and accessing help. This research lays the foundations for future research to consider the mechanisms involved in peer-to-peer connections and the benefits of these in supporting parents and promoting appropriate mental health advice. However, caution has been urged regarding the formation of strong web-based relationships as a dependency for these relationships can exacerbate social withdrawal [46,47]. In addition, as Mumsnet support is not supplemented by professional support, one must consider the consequences of a serious event happening within a supportive group that Mumsnet users are not equipped to deal with, for example, the suicide of a young person discussed in the group. Further research into the disadvantages of such support fora is warranted. For this reason, Mumsnet cannot be a substitute for professional help, and yet thousands of mothers are using the site to receive information and support. Further research should investigate the motivation underlying the use of web-based communities and discussion fora and how they can complement existing professional services.

### Conclusions

In conclusion, the study suggests that parents of children with mental health needs use Mumsnet discussions to offer and consequently receive emotional support and to suggest general advice, techniques, and resources that could be applied outside of help from professional services. In this study, the need for information and advice was particularly apparent for parents who had yet to begin their journey with CAMHS. Overall, this suggests an important role of web-based support for parents looking after young people with mental health needs that warrants further exploration.

## Appendix

Multimedia Appendix 1 Domains, themes and subthemes.

## Abbreviations

CAMHS: Child and Adolescent Mental Health Services
NIHR: National Institute for Health Research
UCL: University College London
