# Supplementary figures and images for: The Use of Mumsnet by Parents of Young People With Mental Health Needs: Qualitative Investigation
Source: JMIR Ment Health. 2020 Sep 3;7(9):e18271. doi: 10.2196/18271 (PMC7499161; doi:10.2196/18271)

Multimedia Appendix 1 : Domains, themes and subthemes.

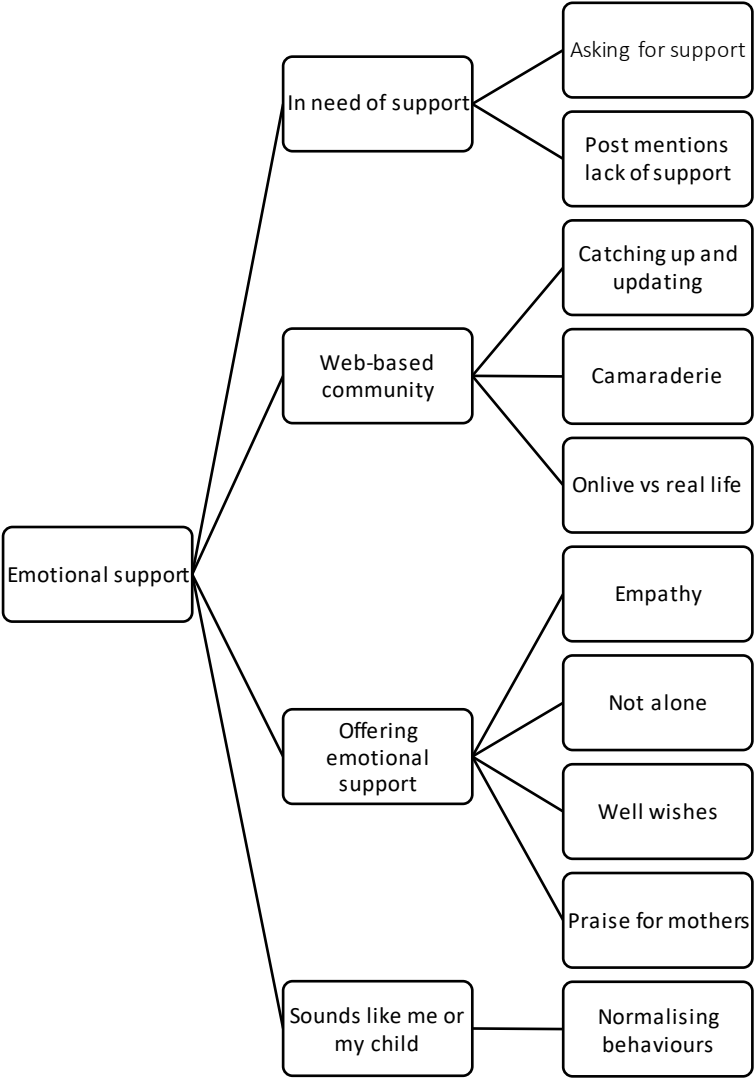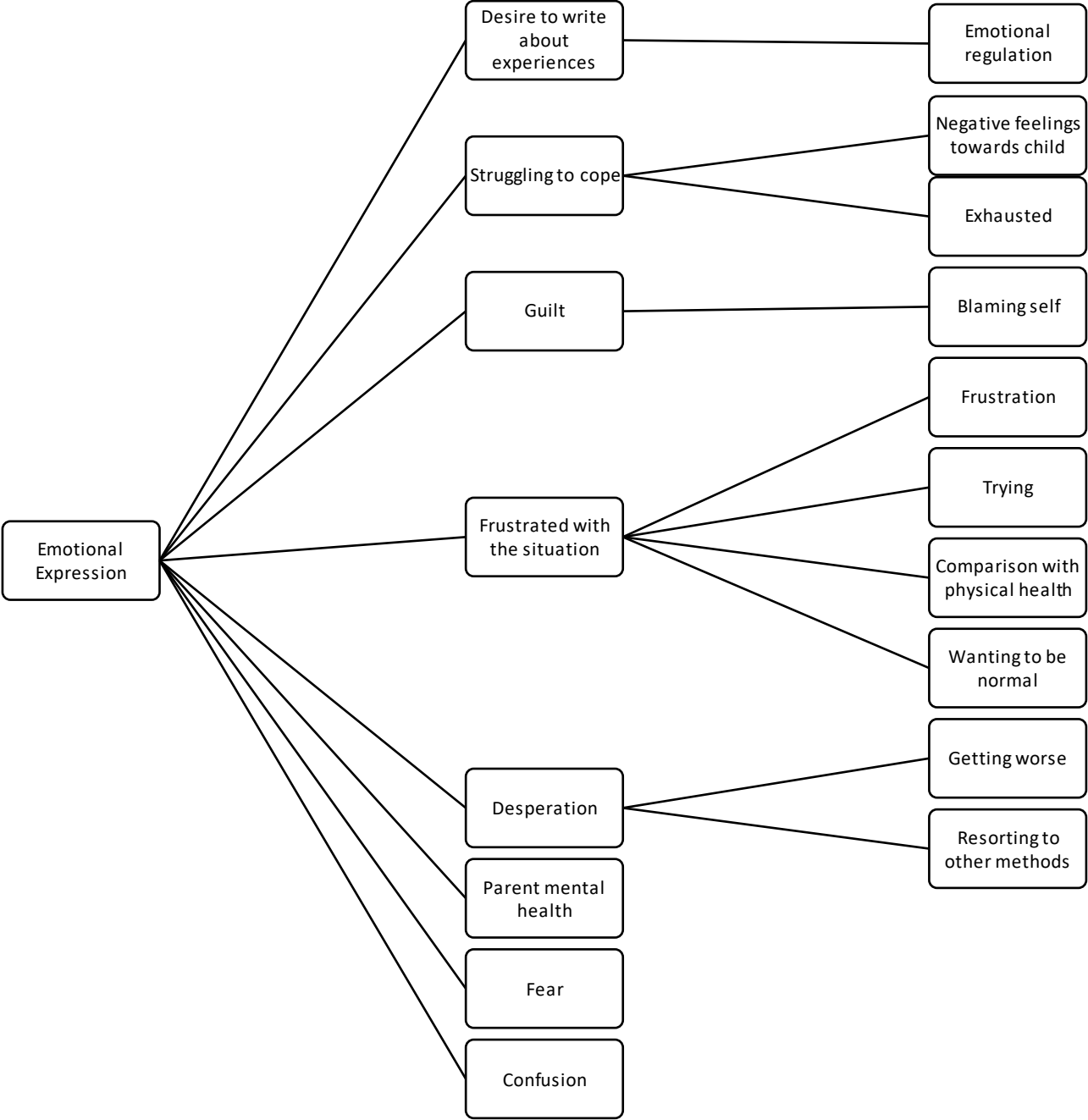

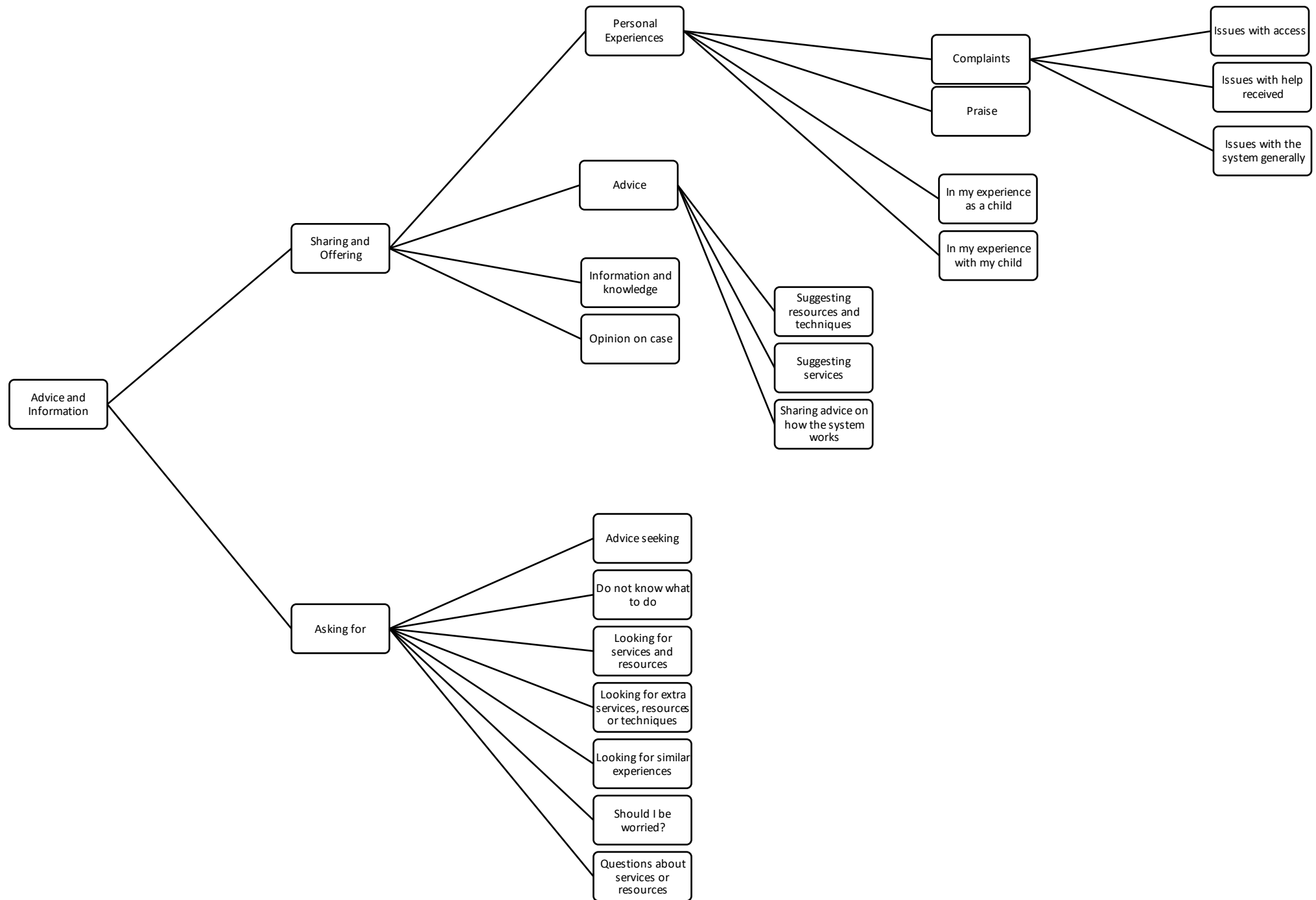

Supplement: Multimedia Appendix 1 [file mental_v7i9e18271_app1.pdf]
